# Broad‐Scale Landscape Structure and Fine‐Scale Microhabitat Jointly Shape the Distribution of the Jeju Salamander, *Hynobius quelpaertensis*

**DOI:** 10.1002/ece3.74661

**Published:** 2026-11-08

**Authors:** Jun‐Sung Kim, Jun‐Kyu Park, Ji‐Eun Lee, Yuno Do

**Affiliations:** ^1^ Department of Biological Sciences Kongju National University Kongju‐si Republic of Korea

**Keywords:** drainage structure, *Hynobius quelpaertensis*, microhabitat, presence‐background model, spatial block cross‐validation, spatial scale, species distribution model

## Abstract

Species distribution models often assume that species occupy all environmentally suitable space, but low‐dispersal amphibians may track landscape structure that has persisted over long periods as well as current local conditions. We examined how broad‐scale landscape structure and fine‐scale microhabitat jointly shape the distribution of the Jeju salamander, *Hynobius quelpaertensis* , on Jeju Island, Republic of Korea. Using 85 spatially thinned occurrence records and 10,000 background points sampled on land, we compared a broad‐scale predictor set (terrain at ≥ 1 km, drainage position and long‐term climate, used as a proxy for a long‐term topographic‐hydrological template) with a fine‐scale set (microtopography, wetness, insolation and canopy). Weighted elastic‐net models were evaluated by spatial block cross‐validation with block‐bootstrap confidence intervals, and a generalized additive model (GAM) with broad‐scale suitability as an offset tested whether microhabitat refined occupancy within broadly suitable space. The broad‐scale and fine‐scale models discriminated occurrences equally well (ROC AUC = 0.79 and 0.78), but the broad‐scale model was better calibrated (Tjur's *R* ^2^ = 0.16 vs. 0.12), and the combined model performed best (ROC AUC = 0.81; Tjur's *R* ^2^ = 0.20). Both sets identified drainage position: occurrence increased with upstream drainage area and local wetness and was higher in hollows and valley bottoms. Within the broad‐scale core, fine‐scale smooth terms doubled Tjur's *R* ^2^ (0.14 to 0.28): occurrence rose with canopy shade and valley depth and declined on exposed south‐facing slopes, although the gain in discrimination was uncertain. Areas suitable only at the fine scale covered 24% of the island but had one‐sixth the presence rate of areas suitable at both scales. Valley drainage structure thus sets the template for the distribution of *H. quelpaertensis* , and shaded, moist microhabitat refines occupancy within it. Conservation should maintain drainage networks together with their forest cover, and candidate unoccupied areas should be surveyed in the field.

## Graphical abstract

This study shows that the distribution of *Hynobius quelpaertensis* on Jeju Island is explained more strongly by a historical topographic‐hydrological template than by contemporary local environmental conditions alone. Historical suitability defined the broad‐scale distributional framework, whereas local microenvironmental variables mainly refined occupancy within the historical core and greatly reduced residual spatial structure. Areas with high contemporary suitability but low historical suitability further suggest the presence of candidate empty niche space consistent with non‐equilibrium distribution dynamics.

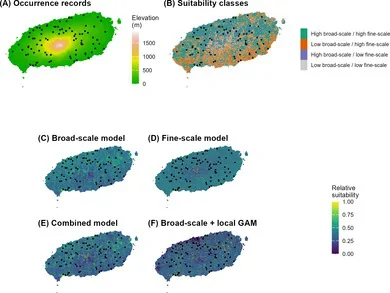

## 1 Introduction

Species distribution models (SDMs) have been widely used to predict the potential distributions of species and to establish conservation priorities. However, many SDM studies rely, either explicitly or implicitly, on the assumption that current occurrence patterns are broadly in equilibrium with present environmental conditions. Such an assumption is not easily met in organisms with low dispersal ability, high habitat fidelity, and strong sensitivity to long‐term environmental stability (Lalechère et al. 2025). In particular, present occurrence may reflect not merely current climate or land cover but also the cumulative effects of past climatic fluctuations, geomorphological history, long‐term hydrological stability, and the accessibility of habitat in the past. Accordingly, attempts to explain present distributions solely in terms of current environmental conditions may oversimplify the actual processes through which distributions are formed (Elith and Leathwick 2009).

Such non‐equilibrium dynamics may be especially pronounced in taxa with limited dispersal and strong dependence on microenvironmental conditions. Amphibians are a representative example, as they are highly sensitive to water loss through the skin, have restricted dispersal capacity, and depend strongly on the microenvironment of both breeding and non‐breeding habitats (Stickley and Fraterrigo 2023). For this reason, the realized distribution of amphibians is unlikely to be explained adequately by broad‐scale contemporary climatic conditions alone, and may instead be shaped simultaneously by long‐maintained moist refugial axes, topographic buffering effects, and local microclimatic conditions. Nevertheless, microclimate or microtopography does not always function as the primary determinant of distribution. Rather, broad‐scale landscape structure that persists over long periods may set the spatial template of a distribution, whereas fine‐scale microhabitat may act as a secondary filter that adjusts local occupancy within it (Seaborn et al. 2025).

These non‐equilibrium distributional responses observed in amphibians are likely to leave particularly clear spatial signatures in salamanders (Um et al. 2025). Because salamanders typically exhibit slow population expansion and high site fidelity to local habitats, their present range may represent not an immediate reflection of current environmental conditions, but the accumulated outcome of past dispersal pathways and long‐term population persistence. Under such circumstances, even environmentally suitable areas at present may remain unoccupied if they were not historically reached or were not occupied continuously over time. Therefore, interpreting salamander distributions requires a non‐equilibrium perspective that considers long‐term topographic and hydrological stability together with historical accessibility, rather than relying only on estimates of present suitability (Peterman and Semlitsch 2014; Bradfer‐Lawrence et al. 2025).

These ideas can be framed within the BAM framework, which distinguishes abiotically suitable conditions (A), biotic interactions (B), and the area that has been accessible to a species through dispersal (M) (Soberon and Peterson 2005). Equilibrium SDMs implicitly assume that occupied space corresponds to the suitable part of M. For low‐dispersal species, however, M may be smaller than the suitable area, so that suitable but inaccessible areas remain unoccupied (Svenning and Skov 2004). Correlative SDMs cannot measure M directly, but comparing predictors that differ in spatial scale and persistence can show whether occupied space is aligned more with long‐lasting landscape structure or with current local conditions, and where the two disagree.

The distribution pattern of the Jeju salamander, *Hynobius quelpaertensis* , provides an appropriate case for examining whether environmentally suitable space is actually occupied, or whether the absence of occupancy in particular areas is attributable to constraints imposed by historical persistence and accessibility. Although this species is widely distributed throughout the southwestern mainland of Korea as well as on Jeju Island, the island serves as an ideal isolated region for verifying these dynamics. Adults of *H. quelpaertensis* live on land and migrate to wetlands to breed (Kim et al. 2021), and terrestrial *Hynobius* depend on moist substrates and cover (Lai and Lue 2013). Genetic data indicate that the Jeju population passed through a founder effect and is now isolated from mainland populations (Suk et al. 2020). In continuous mainland ecosystems, it is difficult to explain the absence of the species in specific areas due to extensive topographical connectivity (Svenning and Skov 2004; Soberon and Peterson 2005). In contrast, Jeju Island provides a clear spatial framework as a closed system with absolute geographical boundaries. Even within its limited area, Jeju Island exhibits substantial variation in volcanic landform formation and degradation, valley development, and the connectivity of drainage systems. In addition, the effects of large‐scale urban and industrial development have been relatively limited, and continuous montane forests and valley drainage systems have remained comparatively well preserved in the island interior. This spatial structure makes it possible to compare, within the same regional extent, the influence of broad‐scale topographic‐hydrological structure with that of fine‐scale microhabitat (Borzée and Min 2021).

To understand the distribution of *H. quelpaertensis* , it is therefore necessary to distinguish landscape structure established over long periods from local conditions that act at present (Zhang et al. 2006). In this study, we grouped predictors by spatial scale and assumed persistence. A broad‐scale set described terrain structure at ≥ 1 km, drainage‐network position, surface water, and long‐term climate, and served as a proxy for a long‐term topographic‐hydrological template; a fine‐scale set described microtopography, local wetness, insolation, and canopy conditions that buffer the microclimate. Because these proxies do not measure past conditions directly, the analysis tests whether the present distribution is consistent with a hierarchy in which broad‐scale structure sets the template and fine‐scale microhabitat adjusts local occupancy within it (Um et al. 2025).

Accordingly, this study addressed three questions. First, how well do broad‐scale landscape structure and fine‐scale microhabitat, separately and together, explain the distribution of *H. quelpaertensis* ? Second, within broad‐scale suitable space, does fine‐scale microhabitat further refine where the species occurs? Third, where do broad‐scale and fine‐scale suitability disagree, and how are these areas related to realized occupancy? We addressed these questions with spatially cross‐validated presence‐background models, aiming to describe the distribution of *H. quelpaertensis* as the outcome of habitat structure acting at two spatial scales and to identify candidate areas for field surveys and conservation.

## 2 Materials and Methods

### 2.1 Occurrence Data and Background Point Construction

The focal species of this study was the Jeju salamander, *H. quelpaertensis* . Occurrence records were obtained from the amphibian and reptile survey data of the 3rd National Ecosystem Survey conducted by the Ministry of Environment of the Republic of Korea. Records from Jeju Island were collected in 2011 and 2013. The 3rd National Ecosystem Survey was designed on a map‐sheet basis across the country and provides a systematic source of regional environmental and biotic information. From this dataset, only occurrence records of *H. quelpaertensis* within Jeju Island were extracted and used as presence‐only data. Because the survey recorded detections without standardized repeat visits, non‐detection could not be interpreted as absence, and survey effort may have varied among map sheets.

A total of 102 occurrence points were initially available from the raw dataset. To reduce the effects of spatial autocorrelation and survey bias, records were spatially thinned to a minimum nearest‐neighbor distance of 500 m with a greedy algorithm implemented in R: records were processed in random order (seed 20,260,309), and a record was retained only if it lay at least 500 m from all previously retained records. As a result, 85 occurrence points were retained for the final analyses. The coordinates of all occurrence points were first standardized in WGS84 and then transformed to the Korean national coordinate reference system (EPSG:5179) for spatial analyses and distance‐based calculations.

The study area was defined as the entire terrestrial area of Jeju Island. Even within its limited extent, Jeju Island exhibits substantial spatial heterogeneity in volcanic landform formation and degradation, valley development, and drainage connectivity, while continuous montane forests and valley‐associated stream systems remain relatively well preserved in the island interior. These characteristics make the island well suited for separating the effects of broad‐scale topographic‐hydrological structure from those of fine‐scale microhabitat within a single regional extent.

Because only presence data were available, background points were used to characterize the environmental conditions available on the island. We sampled 10,000 background points at random (terra::spatSample) from land cells of the 30 m predictor stack, a sample size commonly used in presence‐background models (Barbet‐Massin et al. 2012). Land cells were those where MERIT Hydro upstream drainage area was defined and the MODIS land‐cover class was not water (IGBP Class 17); all 85 occurrence points fell within this land mask. The final analytical dataset therefore consisted of 85 occurrence points and 10,000 background points. These background points were not interpreted as pseudo‐absences; rather, they were used as reference data to compare the relative environmental characteristics of occurrence locations against those available across Jeju Island as a whole, rather than to indicate current non‐occupancy directly.

### 2.2 Environmental Variable Construction

Environmental variables were compiled in Google Earth Engine from publicly available datasets and processed as 30 m spatial layers. Variables were grouped a priori into two sets according to their spatial scale and the expected persistence of the conditions they represent. The broad‐scale set (40 variables) described terrain structure at ≥ 1 km, drainage‐network position, surface water, and long‐term (1958–2024) climate, and was intended as a proxy for a long‐term topographic‐hydrological template. The fine‐scale set (21 variables) described microtopography at ≤ 300 m, local wetness, insolation, vegetation, and canopy conditions, which act locally and can change over shorter periods. Both sets are proxies defined by scale and assumed persistence; neither provides direct palaeoenvironmental, demographic, or genetic evidence. Definitions, data sources, native resolutions, and formulas of all variables are given in Table S2.

Broad‐scale topographic variables were derived from the NASADEM 30 m digital elevation model and included elevation (*elev_m*), topographic relief within a 1 km window (*relief_1km*), the standard deviation of slope within a 1 km window (*slope_sd_1km*), and valley depth within a 1 km window (*valley_depth_1km*, *valley_depth_1km_mean*). Hydrological variables were derived from MERIT Hydro and the JRC Global Surface Water dataset. From MERIT Hydro, upstream drainage area (*upa_km2*) and height above the nearest drainage axis (*hnd_m*) were used. From JRC Global Surface Water, surface‐water occurrence frequency (*water_occurrence*) and seasonality (*water_seasonality*) were extracted, and the distance to persistent surface water (*dist2persistentwater_m*) was calculated; because the JRC dataset maps coastal sea as water, this variable measures the distance to the coast or to inland persistent water, whichever is nearer. In addition, a hydrological persistence index (*hydro_persistence_proxy*) was constructed by integrating upstream contributing area, relative elevation above drainage, and surface‐water occurrence characteristics with fixed a priori weights.

Broad‐scale climate variables were derived from monthly TerraClimate data. For the period 1958–2024, long‐term means and interannual variability were calculated for annual mean temperature, annual precipitation, climatic water deficit, soil moisture, and vapor pressure deficit (VPD). Mean values and variability during the breeding season (January–April) were also calculated. In addition, coefficients of variation (CV) for precipitation and soil moisture were derived, and climate‐stability indices (clim_stab_**) were generated by reversing the sign of variability metrics so that higher values represented greater stability. Because TerraClimate has a native resolution of about 4 km, these variables vary little within the island, and the climate‐stability indices are exact sign‐reversed copies of variability metrics.

Fine‐scale variables were intended to capture local microtopographic structure and microclimatic buffering effects. Using NASADEM, slope (*slope_deg*), northness (*northness*), eastness (*eastness*), topographic position index at 90 m and 300 m scales (*tpi_90m*, *tpi_300m*), and valley depth at 300 m and 250 m scales (*valley_depth_300m*, *valley_depth_250m*) were calculated. In addition, a cold‐air pooling index (*cold_air_pooling*) was calculated as valley depth at 300 m weighted by a north‐facing aspect term, and a solar exposure index (*solar_exposure_proxy*) was calculated from slope and aspect, with high values on steep south‐facing slopes. Local wetness was represented by wetness indices calculated from MERIT Hydro and the DEM (*twi_proxy*, *twi_250m*). The 1 km mean wetness index (*twi_1km*) was assigned to the broad‐scale set.

Vegetation structure and shading effects were represented using the mean and standard deviation of NDVI and EVI from MODIS MOD13Q1 (*ndvi_mean*, *ndvi_sd*, *evi_mean*, *evi_sd*), together with canopy cover from Hansen Global Forest Change (*treecover2000*, *treecover2000_250m*). Land‐cover categories were represented using the IGBP classification from MODIS MCD12Q1 (*lc_type1*), and forest disturbance was represented by *forest_loss_any* and *years_since_loss*. A canopy shade index (*canopy_shade_proxy*) was calculated as the average of mean NDVI and tree‐cover fraction (Table S2).

### 2.3 Environmental Variable Extraction and Construction of the Analytical Dataset

Environmental values at occurrence points were extracted in Google Earth Engine by sampling pixel values at each point location, and values at background points were read from the exported raster stack; the two procedures gave identical values at occurrence locations. In the extracted dataset, the response variable was defined as *label*, with occurrence points coded as 1 and background points coded as 0. Each record also included geographic coordinates, a unique identifier, and the full set of environmental variables.

During construction of the analytical dataset, numerical variables were screened for anomalous values and non‐finite entries. A log1p transformation was applied to variables with strong positive skewness, namely upstream drainage area (*upa_km2*), height above the nearest drainage axis (*hnd_m*), distance to persistent surface water (*dist2persistentwater_m*), and topographic relief (*relief_1km*). The land‐cover variable (*lc_type1*) was treated as a categorical predictor. Because elastic‐net regularization accommodates correlated predictors through shrinkage and selection, pre‐screening was intended only to remove near‐duplicate variables. Within each set, for every numerical predictor pair with |*r*| ≥ 0.97, the variable with the higher mean absolute correlation with the other predictors was removed; six variables were protected a priori (Table S2). This step removed, for example, valley depth at 300 m, which is the exact negative of the topographic position index at 300 m, and the climate‐stability indices, and it retained 24 of 40 broad‐scale and 18 of 21 fine‐scale predictors. Because this threshold is more permissive than commonly recommended (|*r*| > 0.7; Dormann et al. 2013), all broad‐scale models were also refitted after screening at |*r*| ≥ 0.70 and after iterative removal of variables with a variance inflation factor (VIF) ≥ 5 (Figure S1).

For elastic‐net modeling, preprocessing was performed separately within each training set. Numerical predictors were median‐imputed for missing or non‐finite values and then standardized to zero mean and unit variance using training‐set statistics. Categorical predictors were encoded by sparse model matrices, and new data were aligned to the same design‐matrix columns as the corresponding training data before prediction. For the local GAM, missing values in local numerical predictors were likewise replaced by training‐set medians. Smooth terms in the local GAM were specified a priori and were not subject to pre‐screening.

### 2.4 Spatial Cross‐Validation and Sample Weighting

Model evaluation was conducted using spatial block cross‐validation rather than random partitioning. Jeju Island was divided into 5 km × 5 km blocks, and blocks were allocated to five outer folds with a greedy algorithm that balanced the numbers of points and presences among folds, so that spatially adjacent points were assigned to the same fold (Roberts et al. 2017). During the internal tuning stage, four‐fold validation was performed on the training data only, using coordinate‐based k‐means partitioning. This procedure was intended to reduce the risk of overestimating model performance due to spatial autocorrelation.

Because the numbers of occurrence points and background points differed greatly, observation weights were assigned in the elastic‐net models and the local GAM so that the effective prevalence was set to 0.5. Specifically, the weights were adjusted so that occurrence points and background points had the same total weight and were then normalized so that the overall mean weight was 1. Accordingly, the predictions generated in this study were interpreted not as absolute probabilities of occupancy but rather as relative occurrence intensity or relative suitability within a presence‐background framework.

### 2.5 Distribution Modeling

To evaluate broad‐scale distribution structure, we fitted weighted binomial elastic‐net models with glmnet (cv.glmnet; Friedman et al. 2010) to three predictor sets: the broad‐scale set, the fine‐scale set, and both sets combined. Weighted presence‐background logistic regression estimates the relative intensity of occurrence and is closely related to MaxEnt (Fithian and Hastie 2013; Renner and Warton 2013). We used elastic‐net regularization because it accommodates many correlated predictors through shrinkage and automatic variable selection, yields interpretable coefficients for standardized predictors, and produces a linear predictor that can be passed to the second‐stage model as an offset. These models assume additive, linear effects on the logit scale; nonlinear responses were examined in the second stage. To check that the conclusions did not depend on the algorithm, MaxEnt models (maxnet; Phillips et al. 2017) with linear, quadratic, and hinge features and a regularization multiplier of 1 were fitted to the same predictor sets and evaluated on the same folds.

The elastic‐net mixing parameter, α, was evaluated over the candidate set 0, 0.05, 0.10, 0.25, 0.50, 0.75, and 1.00. Within each outer training set, λ was selected by internal cross‐validation using deviance (standardize = FALSE, because predictors had already been standardized), and predictions were generated using *lambda.1se* to reduce overfitting. For each predictor set, fold‐wise predictions were obtained for every α value, and the final α was selected as the value minimizing cross‐validated log loss across the full dataset. After α selection, the three models were refitted using the full dataset.

To evaluate whether fine‐scale microhabitat refined occupancy within broad‐scale suitable space, we constructed a second‐stage local model. Broad‐scale suitability was first obtained from the broad‐scale model, and the broad‐scale core was defined as locations with broad‐scale suitability greater than or equal to the 10th percentile of suitability values at occurrence locations. The local model was then fitted only within this core.

The response variable remained the occurrence/background label. The logit‐transformed broad‐scale suitability was included as a fixed offset, and a weighted binomial generalized additive model (GAM; Wood 2011) was fitted with thin‐plate regression spline smooths (k = 6) for the local wetness index (*twi_proxy*), valley depth at 300 m (*valley_depth_300m*), cold‐air pooling (*cold_air_pooling*), solar exposure (*solar_exposure_proxy*), canopy shading effect (*canopy_shade_proxy*), northness (*northness*), and slope (*slope_deg*). Model fitting was performed using mgcv::bam with method = “fREML”, discrete = TRUE, and select = TRUE; the selection penalty allows a smooth term to be shrunk to zero. This specification assumes that fine‐scale effects are additive on the logit scale, conditional on broad‐scale suitability. Because cold‐air pooling was defined as valley depth weighted by aspect, its partial effect is conditional on valley depth. Local numerical predictors used in this GAM were median‐imputed using training‐set values before prediction.

Local performance was estimated by nested cross‐validation to avoid information leakage. Within each outer training partition, the broad‐scale model was refitted, the core threshold was recalculated, and the GAM was fitted; predictions were then made for core points in the held‐out fold. GAM predictions were compared with two baselines: the offset alone and a recalibrated offset in which only an intercept was estimated. The second baseline separates improvements due to recalibration of the offset from those due to the fine‐scale predictors.

### 2.6 Suitability Classes and Candidate Unoccupied Areas

To examine where broad‐scale and fine‐scale suitability disagree and how this relates to realized occupancy, all points and raster cells were assigned to four classes using thresholds equal to the 10th percentile of each model's predictions at occurrence locations: high broad‐scale and high fine‐scale suitability (Class 1), low broad‐scale and high fine‐scale suitability (Class 2), high broad‐scale and low fine‐scale suitability (Class 3), and low suitability for both (Class 4). Presence rates were compared among classes as the number of occurrence points per 1000 background points.

Class 2 was treated as candidate unoccupied suitable area. Such areas may reflect constraints on access or long‐term persistence, but they may equally reflect incomplete sampling, imperfect detection, unmeasured habitat variables, or disturbance; they are therefore presented as candidates that require field validation.

### 2.7 Model Performance Evaluation, Spatial Diagnostics, and Spatial Prediction

For the broad‐scale, fine‐scale, and combined models, performance was evaluated using fold‐wise predictions from the outer spatial block cross‐validation. Evaluation metrics included ROC AUC, PR AUC, the continuous Boyce index (Hirzel et al. 2006), Brier score, log loss, Tjur's R^2^ (Tjur 2009), calibration intercept, and calibration slope. ROC AUC and PR AUC were computed with pROC (Robin et al. 2011) and PRROC (Grau et al. 2015); PR AUC should be read against its baseline, the presence prevalence (85/10,085 = 0.008). Ninety‐five percent confidence intervals for each metric and for differences between models were obtained by a spatial block bootstrap, in which the 5 km blocks were resampled with replacement 500 times and the metrics were recomputed from the out‐of‐fold predictions. Metrics were also calculated separately for each fold.

Local models were evaluated with the same metrics and bootstrap procedure, using the out‐of‐fold predictions for core points from the nested cross‐validation.

Residual spatial autocorrelation was assessed using Moran's *I* (ape; Paradis and Schliep 2019). For this purpose, up to 800 points were randomly sampled, and an inverse‐distance weighting matrix was constructed among sampled locations. Moran's *I* was calculated for residuals from the broad‐scale models using their fold‐wise predictions and within the core for the GAM and both baselines. This analysis tested whether residual spatial structure remained after model fitting.

Spatial predictions from the final models were generated block by block with terra from the 30 m raster stack and masked to the land mask. Maps were produced for the broad‐scale, fine‐scale, and combined models, for broad‐scale suitability refined by the local GAM within the core, for the core itself, and for the four suitability classes. The analytical workflow is summarized in Figure S2 and Table S1. All analyses were performed in R 4.6.1 (R Core Team 2026); package versions are listed in Table S1.

## 3 Results

### 3.1 Broad‐Scale Distribution Models

The α values selected by cross‐validation were 0.05 for the broad‐scale model, 0 for the fine‐scale model, and 0.10 for the combined model. The broad‐scale and fine‐scale models discriminated occurrence from background points equally well (ROC AUC = 0.787, 95% CI 0.726–0.845, and 0.782, 0.717–0.841; difference = 0.004, −0.030 to 0.038; Figure 2A). The broad‐scale model, however, gave better‐calibrated relative suitability (Tjur's *R* ^2^ = 0.163 vs. 0.122, difference = 0.041, 0.016–0.067; log loss = 0.558 vs. 0.586) and a higher Boyce index (0.929 vs. 0.643), although the Boyce intervals overlapped. The combined model performed best on all metrics (ROC AUC = 0.809, 0.744–0.867; Boyce index = 0.950; Tjur's *R* ^2^ = 0.199, 0.151–0.241; log loss = 0.527; Brier score = 0.170). Its gain over the fine‐scale model was supported for ROC AUC (0.027, 0.004–0.053), and its gain over the broad‐scale model was supported for Tjur's *R* ^2^ (0.035, 0.021–0.050) and log loss (−0.030, −0.039 to −0.020) but not clearly for ROC AUC (0.023, −0.004 to 0.047). PR AUC ranged from 0.051 to 0.062, six to eight times its prevalence baseline of 0.008. Fold‐wise ROC AUC ranged from 0.760 to 0.806 (broad‐scale), 0.747 to 0.814 (fine‐scale), and 0.788 to 0.852 (combined). Suitability maps are shown in Figure 1C–E.

**FIGURE 1:**
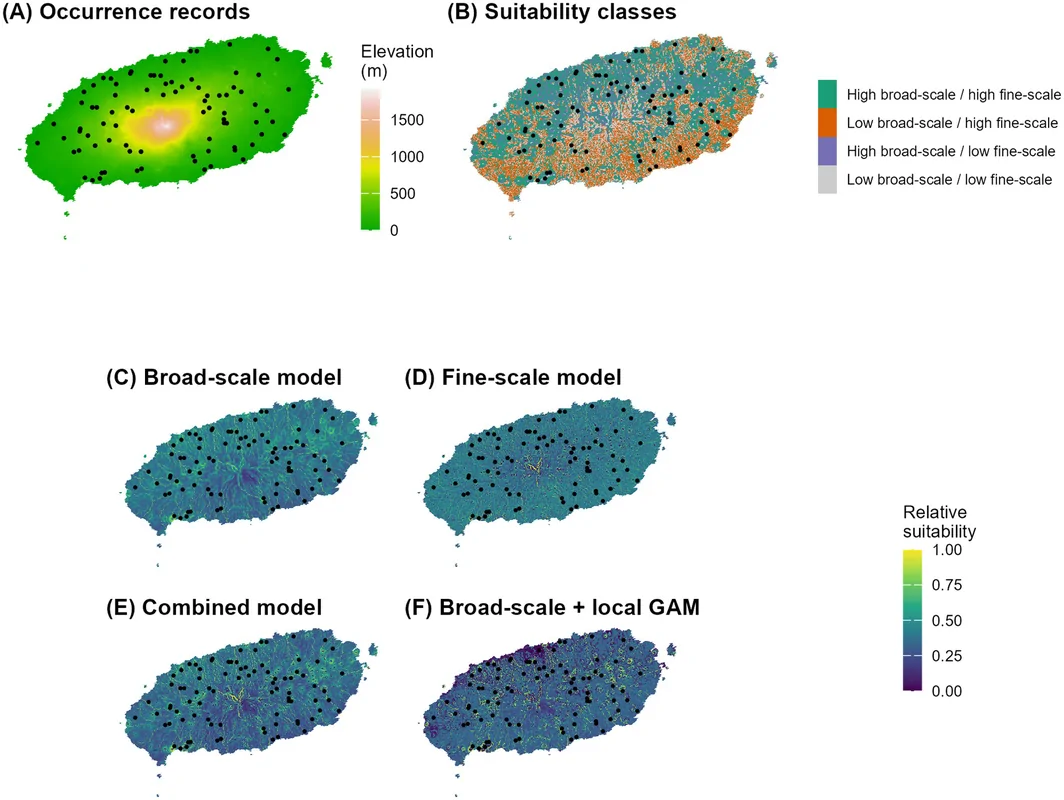
Occurrence records and predicted suitability of *Hynobius quelpaertensis* on Jeju Island. (A) Occurrence records (*n* = 85 after thinning) shown on elevation. (B) Suitability classes from the joint classification of broad‐scale and fine‐scale suitability (thresholds = 10th percentile of predictions at occurrence points); Class 2 (low broad‐scale, high fine‐scale suitability) marks candidate unoccupied suitable areas. (C–E) Relative suitability from the broad‐scale, fine‐scale and combined elastic‐net models. (F) Broad‐scale suitability refined by the local GAM within the broad‐scale core. Black dots indicate occurrence records; all maps are masked to land.

These results were robust to predictor screening and modeling algorithm (Figure S1). With screening at |*r*| ≥ 0.70, ROC AUC was 0.777, 0.771 and 0.806 for the broad‐scale, fine‐scale and combined models, and with VIF < 5 screening it was 0.775, 0.771 and 0.811. In both cases, the broad‐scale and fine‐scale models did not differ in ROC AUC, and the combined model had the highest ROC AUC. MaxEnt models showed the same pattern (ROC AUC = 0.804, 0.801 and 0.825).

### 3.2 Local Refinement Within the Broad‐Scale Core

The broad‐scale core (broad‐scale suitability ≥ 0.376) contained 5514 of the 10,085 points, including 76 of the 85 occurrence points. In nested cross‐validation, adding fine‐scale smooth terms to the broad‐scale offset mainly improved calibration and explained variation within the core (Figure 2B). Tjur's *R* ^2^ increased from 0.133 for the offset alone and 0.138 for the recalibrated offset to 0.283 (difference from the recalibrated offset = 0.146, 0.103–0.192), and log loss decreased from 0.686 and 0.592 to 0.493 (difference = −0.098, −0.124 to −0.072). ROC AUC increased from 0.755 to 0.777, but this gain was uncertain (0.022, −0.031 to 0.078), and PR AUC and the Boyce index did not differ among the three models.

**FIGURE 2:**
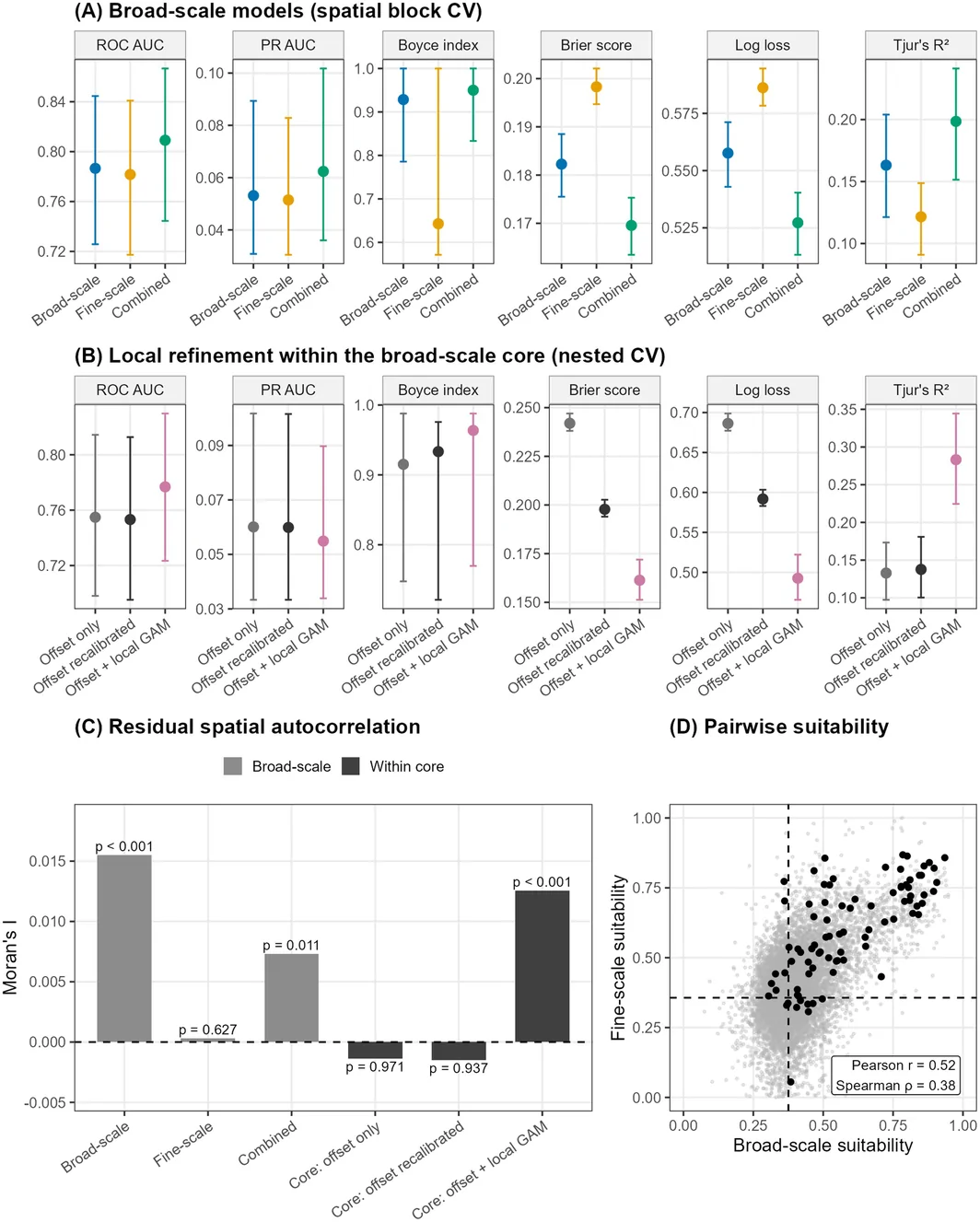
Model performance, residual spatial autocorrelation and the relationship between broad‐scale and fine‐scale suitability. (A) Performance of the broad‐scale, fine‐scale and combined models under spatial block cross‐validation. (B) Performance within the broad‐scale core of the offset‐only baseline, the recalibrated offset and the offset + local GAM model under nested cross‐validation. In (A) and (B), points are estimates from out‐of‐fold predictions and bars are 95% confidence intervals from 500 spatial block‐bootstrap resamples. (C) Moran's *I* of out‐of‐fold residuals, with *p* values. (D) Broad‐scale versus fine‐scale suitability at background (gray) and occurrence (black) points; dashed lines show the class thresholds.

Residual spatial autocorrelation was weak for all models (|Moran's *I*| ≤ 0.016; Figure 2C). It was statistically detectable for the broad‐scale model (*I* = 0.016, *p* < 0.001), the combined model (*I* = 0.007, *p* = 0.011), and the GAM within the core (*I* = 0.013, *p* < 0.001), but not for the fine‐scale model or the two core baselines (*p* ≥ 0.63).

### 3.3 Suitability Classes and Candidate Unoccupied Areas

The class thresholds were 0.376 for broad‐scale suitability and 0.357 for fine‐scale suitability. On land, Class 1 (high suitability at both scales) covered 38.1% of cells, Class 2 (low broad‐scale, high fine‐scale suitability) 24.0%, Class 3 (high broad‐scale, low fine‐scale suitability) 16.6%, and Class 4 (low at both scales) 21.4% (Figure 1B).

Of the 85 occurrence points, 69 fell in Class 1, seven each in classes 2 and 3, and two in Class 4. The presence rate was 18.5 per 1000 background points in Class 1, compared with 3.0 in Class 2, 4.1 in Class 3, and 0.9 in Class 4. Candidate unoccupied areas (Class 2) therefore had about one‐sixth of the presence rate of areas suitable at both scales, and a rate similar to that of Class 3.

### 3.4 Selected Predictors and Local Response Curves

Both single‐set models gave the largest weights to predictors describing drainage position (Figure 3; all coefficients in Table S2). In the broad‐scale model, occurrence increased with upstream drainage area (+0.268) and topographic heterogeneity within 1 km (+0.244) and decreased with height above the nearest drainage (−0.161), followed by the hydrological persistence index (+0.102) and the CV of annual precipitation (−0.079). In the fine‐scale model, occurrence increased with the local wetness index (+0.322) and decreased with the topographic position index at 90 m and 300 m (−0.285 and −0.242), that is, it was higher in hollows and valley bottoms. The combined model retained the leading predictors of both sets: topographic heterogeneity (+0.282), upstream drainage area (+0.271), the topographic position index at 90 m (−0.255) and 300 m (−0.206), and height above the nearest drainage (−0.181).

**FIGURE 3:**
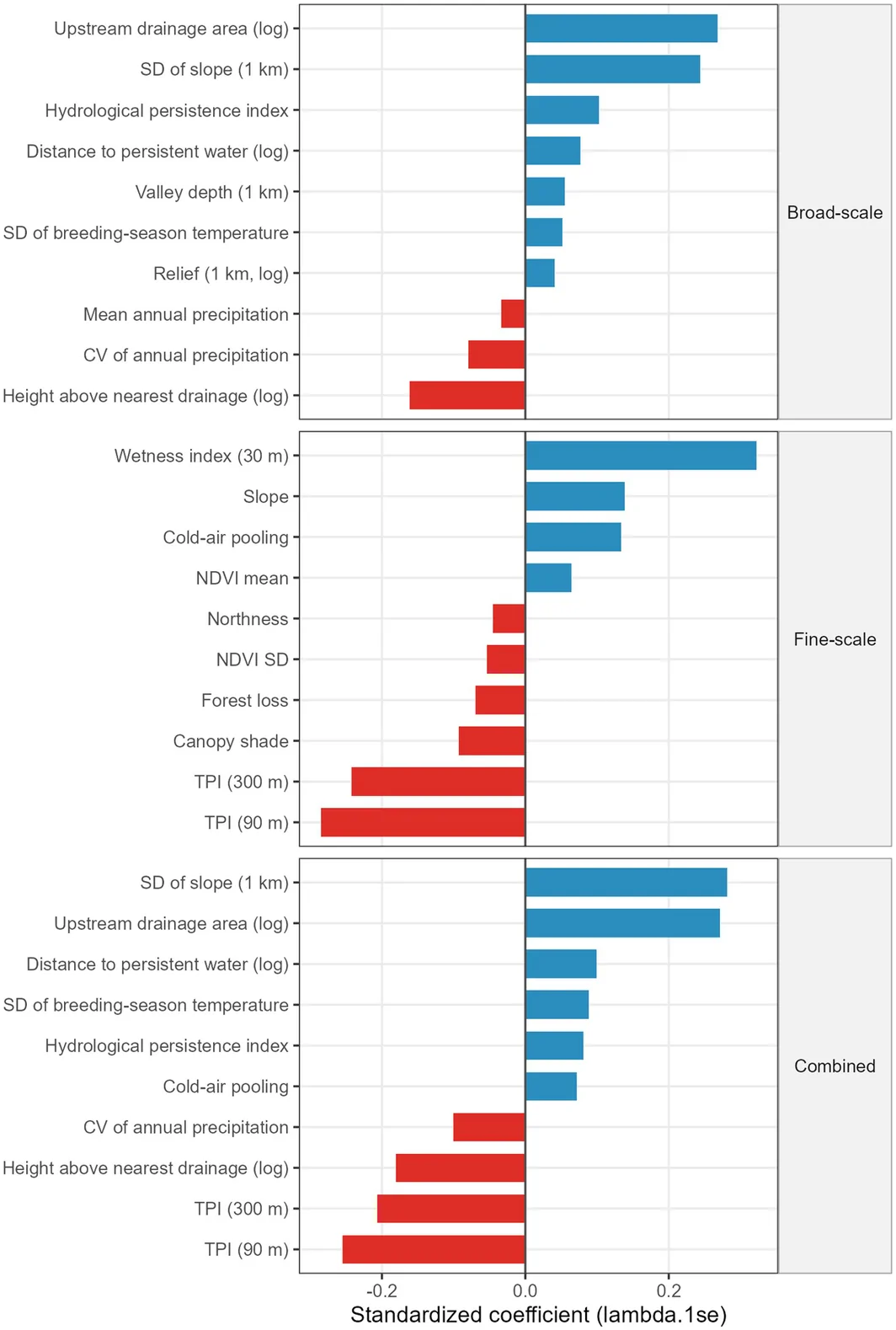
Coefficients of the elastic‐net models. The ten non‐zero standardized coefficients (lambda.1se) with the largest absolute values are shown for the broad‐scale, fine‐scale and combined models (blue, positive; red, negative). All coefficients are listed in Table S2.

Pre‐screening removed 16 broad‐scale predictors (12 TerraClimate variables and the four climate‐stability indices) and three fine‐scale predictors (valley depth at 300 m, tree cover in 2000 and years since forest loss; Table S2).

Within the core, six of the seven smooth terms were retained (all *p* < 0.001), and the GAM explained 33.1% of the deviance (Figure 4). Canopy shade had the strongest effect: occurrence rose steeply as the index increased from about 0.2 to 0.3 and remained higher than at low shade above that value. Occurrence increased almost monotonically with valley depth at 300 m and was highest at intermediate solar exposure, declining on the most exposed, steep south‐facing slopes (index > 0.2). The local wetness index, northness, and cold‐air pooling had smaller nonlinear effects, and slope was shrunk to zero (edf = 0.00, *p* = 0.94).

**FIGURE 4:**
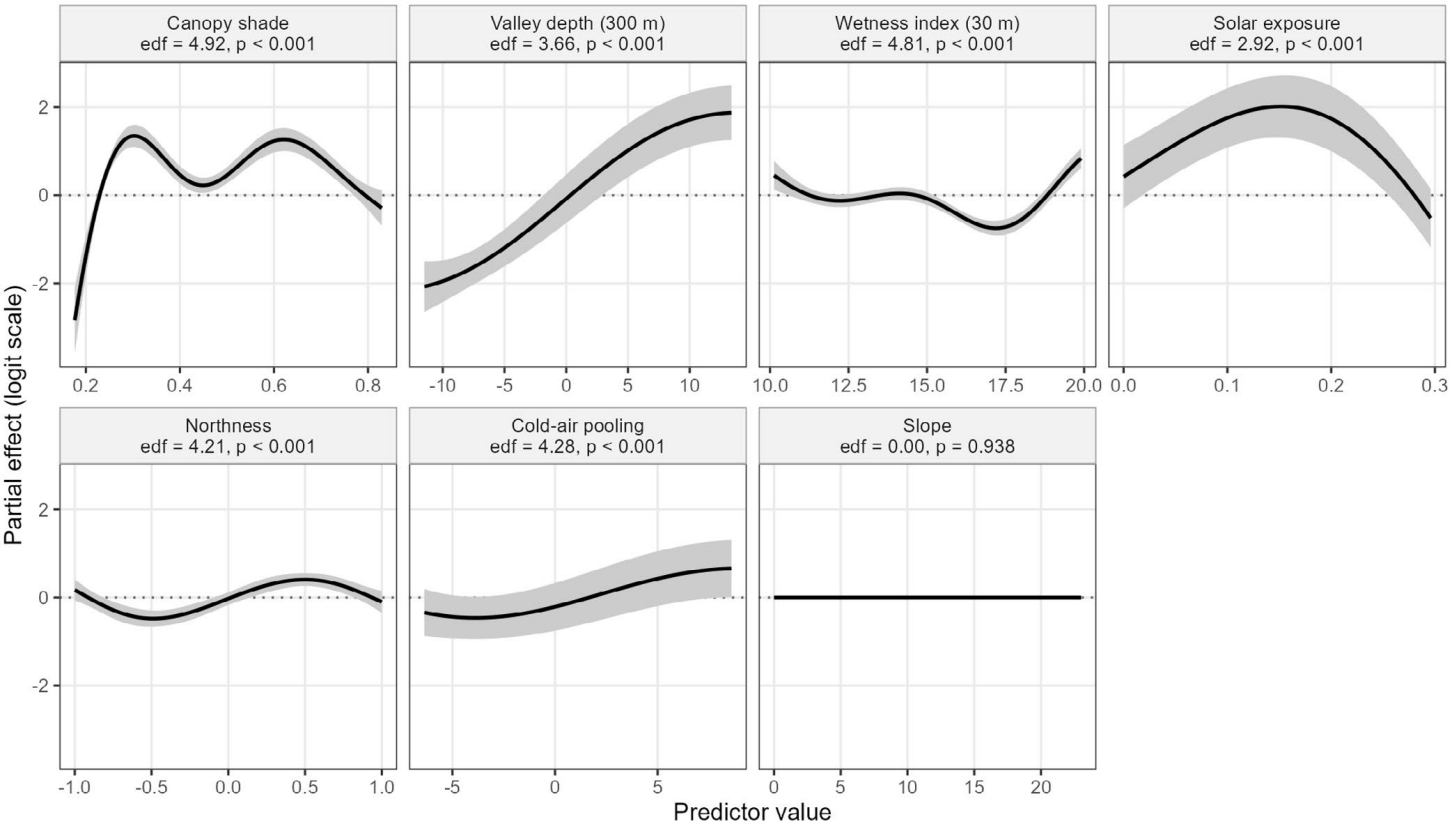
Smooth functions of the local GAM fitted within the broad‐scale core. Partial effects (logit scale) of the seven fine‐scale predictors, conditional on broad‐scale suitability included as an offset and with the other predictors at their medians. Panels are ordered by χ^2^, and edf and *p* values are given for each term. Shaded areas are 95% confidence intervals. Slope was shrunk to zero by the selection penalty.

### 3.5 Relationship Between Broad‐Scale and Fine‐Scale Suitability

Mean broad‐scale, fine‐scale, and combined suitability was 0.591, 0.599, and 0.634 at occurrence points and 0.409, 0.401, and 0.366 at background points.

Broad‐scale and fine‐scale suitability were moderately correlated across all points (Pearson *r* = 0.517; Spearman ρ = 0.381), more strongly at occurrence points (*r* = 0.702; ρ = 0.704) than at background points (*r* = 0.504; ρ = 0.373; Figure 2D).

## 4 Discussion

### 4.1 Complementary Broad‐Scale and Fine‐Scale Structure

The broad‐scale and fine‐scale predictor sets discriminated occurrence from background points equally well, and the combined model performed best. The distribution of *H. quelpaertensis* on Jeju Island is therefore better described as the joint outcome of broad‐scale landscape structure and fine‐scale microhabitat than as the product of either alone (Bean et al. 2014). The broad‐scale set nevertheless carried additional information on relative suitability: its predictions were better calibrated and had a higher Boyce index than those of the fine‐scale set, and the gain of the combined model over the broad‐scale model lay mainly in calibration rather than discrimination. These patterns were consistent across predictor‐screening rules and between elastic‐net and MaxEnt models (Figure S1).

Both sets pointed to the same landscape feature, drainage position. At the broad scale, occurrence was associated with large upstream drainage area, low height above the nearest drainage, and high topographic heterogeneity; at the fine scale, it was associated with high local wetness and low topographic position, that is, with hollows and valley bottoms. This is consistent with the ecology of *Hynobius*: adults of *H. quelpaertensis* live on land but migrate to wetlands to breed (Kim et al. 2021), and terrestrial *Hynobius* favor moist substrates and cover objects (Lai and Lue 2013). Pond‐ and stream‐breeding salamanders typically use terrestrial habitat within a few hundred meters of their breeding sites (Semlitsch and Bodie 2003). Valley‐bottom drainage networks combine moist terrestrial habitat with access to breeding water, which may explain why the same structure emerged at both scales.

The broad‐scale set was intended as a proxy for a long‐term topographic‐hydrological template, and drainage networks and valley structure on a volcanic island persist over long periods. The calibration advantage of the broad‐scale set is consistent with a role for such persistent structure, as are the founder effect and the island‐mainland isolation inferred from genetic data (Suk et al. 2020). However, the broad‐scale variables are present‐day measurements, and the near‐equal discrimination of the two sets shows that this proxy cannot separate historical from contemporary influences. Testing whether past accessibility or persistence constrains the present distribution will require direct evidence, such as fine‐scale population genetic structure (Suk et al. 2020) or palaeoenvironmental reconstructions (Marta et al. 2023).

### 4.2 Refinement by Fine‐Scale Microhabitat

Within the broad‐scale core, fine‐scale microhabitat refined relative suitability. Adding fine‐scale smooth terms doubled Tjur's *R* ^2^ and reduced log loss beyond what recalibration of the broad‐scale offset achieved, although the gain in ROC AUC was small and uncertain. Fine‐scale variables therefore improved how suitability was graded among locations that were already broadly suitable more than they improved the ranking of occupied and unoccupied locations. This is the pattern expected if broad‐scale structure sets the template and microhabitat modulates conditions within it (Seaborn et al. 2025).

Residual spatial autocorrelation was weak in all models (|Moran's *I*| ≤ 0.016). Some of these small values were statistically detectable because of the large sample, but they are unlikely to have affected the model comparisons, which were based on spatial block cross‐validation.

The shapes of the response curves are consistent with the water balance of salamanders, which lose water rapidly through permeable skin. Occurrence rose sharply with canopy shade up to an index of about 0.3, suggesting avoidance of open, unshaded ground, as reported for salamanders near clear‐cut edges (deMaynadier and Hunter 1998). Occurrence also increased with valley depth and declined on the most exposed, steep south‐facing slopes, where insolation and evaporative demand are highest (Peterman and Semlitsch 2014). The cold‐air pooling term should be read with care, because it is valley depth weighted by aspect, so its effect is conditional on valley depth itself. Together, these effects describe shaded, moist valley habitat within the broad drainage template, consistent with microhabitat studies of other salamanders (Ficetola et al. 2018; Stickley and Fraterrigo 2023; Yeiser and Richter 2015).

### 4.3 Candidate Unoccupied Suitable Areas

Occurrences were concentrated where both broad‐scale and fine‐scale suitability were high, and areas suitable only at the fine scale (Class 2) had a presence rate about one‐sixth as high. These candidate unoccupied areas covered about a quarter of the island.

Several explanations are possible. Class 2 areas may be less accessible from occupied drainages or less able to support persistent populations, in line with the limited dispersal of many amphibians (Smith and Green 2005; Lalechère et al. 2025). They may equally reflect incomplete survey coverage, imperfect detection in presence‐only data, unmeasured factors such as the availability of breeding water, or recent disturbance. Because these explanations cannot be separated with the present data, Class 2 areas are best treated as targets for field surveys rather than as evidence of unoccupied niche space.

For conservation, the results suggest that protecting *H. quelpaertensis* on Jeju requires maintaining habitat at both scales: valley drainage networks and the shaded, moist forest within them. Areas with high suitability at both scales, which held 69 of the 85 records, are the clearest priorities, and connectivity along drainages may be important for linking them (Luo et al. 2021; Matos et al. 2019). Field surveys of Class 2 areas would show whether they are unoccupied habitat or undersampled populations.

It should be noted that the scope of this study is limited to the Jeju Island population. Since *H. quelpaertensis* is generally known to be widely distributed not only on Jeju Island but also in the southwestern region of the Korean Peninsula (Baek et al. 2011; Min et al. 2016), the spatial patterns of suitability observed in this study may not fully explain the species' total distribution range. Island ecosystems may exhibit fundamentally different geomorphological, hydrological, and spatial structures compared to the mainland (Weigelt et al. 2013; Whittaker et al. 2017), which can lead to different patterns in the relationship between broad‐scale landscape structure and fine‐scale microhabitat. Therefore, future research should extend this analytical approach to mainland populations to conduct a comparative analysis of how landscape structure, microhabitat, and population history influence actual distribution and conservation priorities in each population.

Several further limitations should be noted. The occurrence data are presence‐only records from surveys in 2011 and 2013 without repeat visits, so detection probability and survey effort could not be modeled (Freeman et al. 2022), and the predictors refer to different periods (e.g., climate for 1958–2024 and land cover for the most recent year). Several predictors are derived proxies with fixed weights (Table S2), and TerraClimate variables vary little within the island because of their coarse resolution. Finally, PR AUC values were low in absolute terms (0.05–0.06), as expected for a rare species evaluated against 10,000 background points, although they were six to eight times the prevalence baseline.

## 5 Conclusion

The distribution of *H. quelpaertensis* on Jeju Island reflects habitat structure at two spatial scales. Broad‐scale and fine‐scale predictors discriminated occurrences equally well, both identified drainage position as the main correlate of occurrence, and models combining them performed best. Within broadly suitable space, shaded, moist valley microhabitat further refined relative suitability. Areas suitable only at the fine scale were rarely occupied, but whether this reflects limited access, unmeasured habitat requirements, or incomplete sampling remains to be tested. Because the broad‐scale predictors are present‐day proxies of long‐term landscape structure, future studies should test historical constraints directly with population genetic and palaeoenvironmental data, and should extend this framework to mainland populations.

## Author Contributions

**Jun‐Sung Kim:** data curation (equal), formal analysis (equal), visualization (equal), writing – original draft (lead). **Jun‐Kyu Park:** data curation (equal), formal analysis (equal), visualization (equal), writing – review and editing (equal). **Ji‐Eun Lee:** data curation (equal), formal analysis (equal), writing – review and editing (equal). **Yuno Do:** conceptualization (lead), funding acquisition (lead), methodology (lead), project administration (lead), resources (lead), supervision (lead), validation (lead), writing – original draft (equal), writing – review and editing (equal).

## Funding

This work was supported by the National Research Foundation of Korea, RS‐2025‐1606666030782064780001.

## Conflicts of Interest

The authors declare no conflicts of interest.

## Supporting information


**Table S1:** Summary of the analytical workflow used to compare broad‐scale (historical template) and fine‐scale (contemporary local‐filter) predictors of the distribution of *Hynobius quelpaertensis* on Jeju Island.

**Table S2:** Candidate predictor variables used to model the distribution of *Hynobius quelpaertensis* on Jeju Island.

**Figure S1:** Sensitivity of model performance to predictor screening and modeling algorithm. ROC AUC and continuous Boyce index for the broad‐scale, fine‐scale and combined models fitted with elastic net (circles) and MaxEnt (triangles) after screening at |*r*| ≥ 0.97 (main analysis), |*r*| ≥ 0.70 or VIF < 5. Points are out‐of‐fold estimates and bars are 95% spatial block‐bootstrap confidence intervals. The right‐hand group shows results with the original background sample, which included sea cells and is superseded.

**Figure S2:** Analytical workflow. Occurrence records and Google Earth Engine predictors were pre‐processed into an analytical dataset of 85 presences and 10,000 land background points, evaluated by 5 km spatial block cross‐validation. Stage 1 compared broad‐scale, fine‐scale and combined elastic‐net models; Stage 2 tested whether fine‐scale microhabitat refined broad‐scale suitability within the broad‐scale core, using a GAM with broad‐scale suitability as an offset. All performance metrics were computed from out‐of‐fold predictions with 95% spatial block‐bootstrap confidence intervals.

## Data Availability

Occurrence‐derived data, the Google Earth Engine and R code, and all result files are available from the Zenodo repository: https://doi.org/10.5281/zenodo.23635896 (Kim et al. 2026).
