# Supplementary material for: Broad‐Scale Landscape Structure and Fine‐Scale Microhabitat Jointly Shape the Distribution of the Jeju Salamander, Hynobius quelpaertensis
Source: Ecol Evol. 2026 Nov 8;16(11):e74661. doi: 10.1002/ece3.74661 (PMC13744882; doi:10.1002/ece3.74661)
Supplement: Supplementary file 1 [file ECE3-16-e74661-s001.docx]

**Table S1. Summary of the analytical workflow used to compare broad-scale (historical template) and fine-scale (contemporary local-filter) predictors of the distribution of *Hynobius quelpaertensis* on Jeju Island**

| Category | Description |
| --- | --- |
| Occurrence data | Occurrence records of *H. quelpaertensis* (n = 102) were obtained from the amphibian and reptile surveys of the 3rd National Ecosystem Survey (Ministry of Environment, Republic of Korea; surveys conducted in 2011 and 2013), which samples the country on a map-sheet basis. Coordinates were converted from WGS84 to EPSG:5179. Records were thinned with a greedy algorithm written in R: records were processed in random order (seed 20260309) and a record was kept only if it lay ≥ 500 m from every record already kept, leaving 85 presences. |
| Background data | A total of 10,000 background points were drawn at random (terra::spatSample) from the 30 m land cells of the predictor stack. Land was defined as cells where MERIT Hydro upstream drainage area is defined and the MODIS IGBP land-cover class is not 17 (water); all 85 presences fell within this mask. Background points describe the environmental conditions available on the island and were not treated as pseudo-absences. |
| Environmental predictors | Sixty-one candidate predictors (Table S2) were derived in Google Earth Engine from NASADEM, MERIT Hydro, JRC Global Surface Water, TerraClimate (1958–2024), MODIS MOD13Q1 and MCD12Q1, and Hansen Global Forest Change, and exported as a 30 m raster stack (EPSG:4326). They were grouped a priori by spatial scale and assumed temporal persistence into a broad-scale set (historical template; 40 variables: terrain structure at ≥ 1 km, drainage-network position, surface water and long-term climate means and variability) and a fine-scale set (contemporary local filter; 21 variables: microtopography at ≤ 300 m, wetness, insolation, vegetation, canopy and land cover). Neither set is a direct palaeoenvironmental, demographic or genetic measure. |
| Analytical dataset construction | Covariate values were extracted at all points. Upstream drainage area, height above nearest drainage, distance to persistent water and 1 km relief were log1p-transformed, and land cover was treated as a categorical predictor. Within each set, predictor pairs with \|r\| ≥ 0.97 were pruned by removing the variable with the higher mean absolute correlation (protected variables in Table S2), which retained 24 of 40 broad-scale and 18 of 21 fine-scale predictors. Within each training partition, missing values were replaced by training medians and numeric predictors were standardized with training means and SDs. Sensitivity analyses used \|r\| ≥ 0.70 and VIF < 5 screening (Fig. S1). |
| Spatial cross-validation and weighting | Points were assigned to 5 km × 5 km blocks (EPSG:5179), and blocks were allocated to five outer folds so that the numbers of points and presences were balanced. Hyperparameters were tuned only within training data, using four-fold k-means clustering of coordinates. Observation weights gave presences and background points equal total weight (effective prevalence 0.5) and were rescaled to a mean of 1, so predictions represent relative suitability rather than probability of occurrence. |
| Broad-scale distribution analysis | Weighted binomial elastic-net models (glmnet::cv.glmnet, standardize = FALSE) were fitted separately to the broad-scale, fine-scale and combined predictor sets. The mixing parameter α was chosen from {0, 0.05, 0.10, 0.25, 0.50, 0.75, 1} by out-of-fold log loss, and λ was set to lambda.1se from inner cross-validation on binomial deviance; α = 1 (lasso) was selected for all three sets. Final models were refitted to the full dataset. As an algorithm check, MaxEnt models (maxnet; linear, quadratic and hinge features, regularization multiplier 1) were evaluated on the same folds (Fig. S1). |
| Local-filter analysis | The broad-scale core was defined as locations whose broad-scale suitability was ≥ the 10th percentile of predictions at presences. Within the core, a weighted binomial GAM (mgcv::bam; method = "fREML", discrete = TRUE, select = TRUE; thin-plate smooths, k = 6) was fitted with logit(broad-scale suitability) as an offset and smooth terms for seven fine-scale variables (Table S2, set L). In nested cross-validation, the broad-scale model, the core threshold and the GAM were refitted within each outer training partition. The GAM was compared with the offset-only baseline and with a recalibrated baseline in which only the intercept was re-estimated. |
| Suitability classes | All points and raster cells were assigned to four classes according to whether broad-scale and fine-scale suitability were at or above the 10th percentile of their values at presences. Class 2 (low broad-scale, high fine-scale suitability) identifies candidate unoccupied suitable areas, and presence rates per 1,000 background points were compared among classes. Such candidates require field validation, because they may also reflect incomplete sampling, imperfect detection or unmeasured habitat factors. |
| Model evaluation | Out-of-fold predictions were evaluated with ROC AUC (pROC), PR AUC (PRROC; prevalence baseline = 0.008), the continuous Boyce index (10 equal-width bins), Brier score, log loss, Tjur’s *R*^2^, calibration intercept and calibration slope. Ninety-five percent confidence intervals for each metric and for between-model differences were obtained from 500 spatial block-bootstrap resamples of the 5 km blocks; fold-wise metrics are also reported. |
| Spatial diagnostics | Spatial autocorrelation of out-of-fold residuals (observed − predicted) was measured with Moran’s *I* (ape::Moran.I; 800 randomly sampled points, inverse-distance weights) for the broad-scale models and, within the core, for the baseline and GAM predictions. |
| Spatial prediction | Final models were applied block by block to the 30 m stack with terra, and all maps were masked to the land mask. Maps show broad-scale, fine-scale and combined suitability, broad-scale suitability refined by the local GAM within the core, the core itself and the four suitability classes. |
| Software and reproducibility | Analyses were run in R 4.6.1 with data.table 1.18.6.1, dplyr 1.2.1, sf 1.1-3, terra 1.9-50, glmnet 5.1, Matrix 1.7-5, mgcv 1.9-4, pROC 1.19.1, PRROC 1.4, ape 5.8-1 and maxnet 0.1.4. The Google Earth Engine extraction script, the R analysis and figure scripts and the result files are archived on Zenodo (Kim et al., 2026). |
