# Supplementary material for: Broad‐Scale Landscape Structure and Fine‐Scale Microhabitat Jointly Shape the Distribution of the Jeju Salamander, Hynobius quelpaertensis
Source: Ecol Evol. 2026 Nov 8;16(11):e74661. doi: 10.1002/ece3.74661 (PMC13744882; doi:10.1002/ece3.74661)
Supplement: Supplementary file 2 [file ECE3-16-e74661-s004.docx]

**Table S2. Candidate predictor variables used to model the distribution of *Hynobius quelpaertensis* on Jeju Island**

All 61 candidate predictors were derived in Google Earth Engine from public datasets and exported at 30 m. The table gives each predictor's definition, source, transformation and its status in the main analysis (land-only background, n = 85 presences + 10,000 background points).

**Set:** H = broad-scale / historical-template set; C = fine-scale / contemporary local-filter set; L = also used as a smooth term in the local GAM. **Status:** Removed = dropped by pre-screening (|r| ≥ 0.97 within its set); Retained = entered the elastic-net model. **β** = non-zero standardized coefficient at lambda.1se in the model of its own set / in the combined model; 0 = shrunk to zero.

**1. Terrain (NASADEM, NASA/NASADEM_HGT/001; 1 arc-second ≈ 30 m; static, SRTM 2000)**

| Code | Set | Description (unit) | Derivation in GEE | Transform | Status | β (own; combined) |
| --- | --- | --- | --- | --- | --- | --- |
| elev_m | H | Elevation (m) | NASADEM elevation band | – | Retained | 0; 0 |
| relief_1km | H | Local relief within 1 km (m) | Focal max − focal min of elevation, circular kernel r = 1 km | log1p | Retained | 0; 0 |
| slope_sd_1km | H | Topographic heterogeneity (°) | Focal SD of slope, r = 1 km | – | Retained | +0.313; +0.316 |
| valley_depth_1km | H | Valley depth at 1 km (m) | Focal mean elevation (r = 1 km) − elevation | – | Retained | 0; 0 |
| valley_depth_1km_mean | H | Smoothed valley depth (m) | Focal mean (r = 1 km) of valley_depth_300m | – | Retained | 0; 0 |
| slope_deg | C, L | Slope (°) | ee.Terrain.products | – | Retained | 0; 0 |
| northness | C, L | Northness (−1 to 1) | cos(aspect) | – | Retained | 0; 0 |
| eastness | C | Eastness (−1 to 1) | sin(aspect) | – | Retained | 0; 0 |
| tpi_90m | C | Topographic position index, 90 m (m) | Elevation − focal mean elevation (r = 90 m); < 0 = hollow | – | Retained | −0.347; −0.328 |
| tpi_300m | C | Topographic position index, 300 m (m) | Elevation − focal mean elevation (r = 300 m) | – | Retained | −0.232; −0.212 |
| valley_depth_300m | C, L | Valley depth at 300 m (m) | Focal mean elevation (r = 300 m) − elevation (= −tpi_300m) | – | Removed (r = −1 with tpi_300m); used in local GAM | – |
| valley_depth_250m | C | Smoothed valley depth (m) | Focal mean (r = 250 m) of valley_depth_300m | – | Retained | 0; 0 |
| cold_air_pooling | C, L | Cold-air pooling proxy (m) | valley_depth_300m × (northness + 1) / 2 | – | Retained | 0; 0 |
| solar_exposure_proxy | C, L | Terrain solar exposure (dimensionless; 0 = flat or north-facing) | (slope / 90) × (1 − northness); high = steep south-facing | – | Retained | 0; 0 |

In the local GAM, valley depth (300 m), cold-air pooling, solar exposure and northness had edf = 3.68, 4.79, 2.88 and 4.32 (all p < 0.001); slope was shrunk to zero (edf = 0.00, p = 0.79).

**2. Hydrology and surface water (MERIT Hydro v1.0.1, 3 arc-second ≈ 90 m; JRC Global Surface Water v1.4, 30 m, 1984–2021)**

| Code | Set | Description (unit) | Derivation in GEE | Transform | Status | β (own; combined) |
| --- | --- | --- | --- | --- | --- | --- |
| upa_km2 | H | Upstream drainage area (km²) | MERIT Hydro upa | log1p | Retained | +0.338; +0.325 |
| hnd_m | H | Height above nearest drainage (m) | MERIT Hydro hnd | log1p | Retained | −0.191; −0.170 |
| water_occurrence | H | Surface-water occurrence (%) | JRC occurrence; 0 where never water | – | Retained | 0; 0 |
| water_seasonality | H | Surface-water seasonality (months yr⁻¹) | JRC seasonality; 0 where never water | – | Retained | 0; 0 |
| dist2persistentwater_m | H | Distance to persistent water (m) | Euclidean distance to pixels with occurrence ≥ 50% or seasonality ≥ 10 months; search 512 px (≈ 15 km); 9999 beyond. Includes the sea (see Notes) | log1p | Retained | 0; 0 |
| hydro_persistence_proxy | H | Hydrological persistence index | 0.5 × occurrence + 5 × seasonality + 10 × ln(upa + 1) − 0.2 × hnd (fixed ad hoc weights) | – | Retained | 0; 0 |
| twi_1km | H | Wetness index, 1 km mean | Focal mean (r = 1 km) of twi_proxy | – | Retained | 0; 0 |
| twi_proxy | C, L | Topographic wetness index | ln(upa × 10⁶ + 1) − ln(tan(slope)); tan(slope) floored at 0.001 | – | Retained | +0.293; 0 |
| twi_250m | C | Wetness index, 250 m mean | Focal mean (r = 250 m) of twi_proxy | – | Retained | 0; 0 |

In the local GAM, twi_proxy had edf = 4.63 (p < 0.001). upa_km2 and hnd_m are undefined over the sea, so MERIT Hydro also defines the land mask (Section 5).

**3. Long-term climate (TerraClimate, IDAHO_EPSCOR/TERRACLIMATE; 1/24° ≈ 4 km; monthly, 1958–2024)**

Annual values were first computed for each of the 67 years: mean temperature = mean of (tmmn + tmmx) / 2 (°C); precipitation (pr) and climatic water deficit (def) = annual sums (mm); soil moisture (soil, mm) and vapour pressure deficit (vpd, kPa) = annual means. Breeding-season (January–April) values were computed the same way over those four months. Each variable is then the mean (_mean), SD (_sd) or SD / mean (_cv) of the 67 annual values. All are in set H; none is transformed.

| Code | Description | Status | β (own; combined) |
| --- | --- | --- | --- |
| tc_tmean_ann_mean | Mean annual temperature (°C) | Retained | 0; 0 |
| tc_tmean_ann_sd | Interannual SD of annual temperature (°C) | Retained | 0; 0 |
| tc_pr_ann_mean | Mean annual precipitation (mm) | Retained | 0; 0 |
| tc_pr_ann_sd | Interannual SD of annual precipitation (mm) | Removed | – |
| tc_pr_ann_cv | CV of annual precipitation | Retained | −0.028; −0.102 |
| tc_def_ann_mean | Mean annual climatic water deficit (mm) | Retained | 0; 0 |
| tc_def_ann_sd | Interannual SD of water deficit (mm) | Removed | – |
| tc_soil_ann_mean | Mean annual soil moisture (mm) | Retained | 0; 0 |
| tc_soil_ann_sd | Interannual SD of soil moisture (mm) | Retained | 0; 0 |
| tc_soil_ann_cv | CV of annual soil moisture | Removed | – |
| tc_vpd_ann_mean | Mean annual VPD (kPa) | Removed | – |
| tc_vpd_ann_sd | Interannual SD of VPD (kPa) | Removed | – |
| tc_tmean_breed_mean | Mean breeding-season temperature (°C) | Removed | – |
| tc_tmean_breed_sd | Interannual SD of breeding-season temperature (°C) | Retained | 0; +0.012 |
| tc_pr_breed_mean | Mean breeding-season precipitation (mm) | Removed | – |
| tc_pr_breed_sd | Interannual SD of breeding-season precipitation (mm) | Retained | 0; 0 |
| tc_pr_breed_cv | CV of breeding-season precipitation | Retained | 0; 0 |
| tc_def_breed_mean | Mean breeding-season water deficit (mm) | Retained | 0; 0 |
| tc_def_breed_sd | Interannual SD of breeding-season water deficit (mm) | Removed | – |
| tc_soil_breed_mean | Mean breeding-season soil moisture (mm) | Removed | – |
| tc_soil_breed_sd | Interannual SD of breeding-season soil moisture (mm) | Removed | – |
| tc_soil_breed_cv | CV of breeding-season soil moisture | Removed | – |
| tc_vpd_breed_mean | Mean breeding-season VPD (kPa) | Retained | 0; 0 |
| tc_vpd_breed_sd | Interannual SD of breeding-season VPD (kPa) | Removed | – |
| clim_stab_tmean | Temperature stability = −tc_tmean_ann_sd | Removed (exact sign-reversed duplicate) | – |
| clim_stab_pr | Precipitation stability = −tc_pr_ann_cv | Removed (duplicate) | – |
| clim_stab_soil | Soil-moisture stability = −tc_soil_ann_cv | Removed (duplicate) | – |
| clim_stab_vpd | VPD stability = −tc_vpd_ann_sd | Removed (duplicate) | – |

Only about 100 TerraClimate cells (≈ 4.6 × 3.9 km each) cover Jeju Island, so these layers vary little at 30 m and most were collinear (16 of 28 removed).

**4. Vegetation, land cover and microclimate proxies (MODIS MOD13Q1 v061, 250 m, 16-day, 2001–2024; Hansen Global Forest Change v1.12, 30 m, 2000–2024; MODIS MCD12Q1 v061, 500 m, most recent year available)**

| Code | Set | Description (unit) | Derivation in GEE | Transform | Status | β (own; combined) |
| --- | --- | --- | --- | --- | --- | --- |
| ndvi_mean | C | Mean NDVI | Mean of all MOD13Q1 NDVI composites × 0.0001 | – | Retained | 0; 0 |
| ndvi_sd | C | SD of NDVI | SD over composites | – | Retained | 0; 0 |
| evi_mean | C | Mean EVI | As ndvi_mean, EVI band | – | Retained | 0; 0 |
| evi_sd | C | SD of EVI | As ndvi_sd, EVI band | – | Retained | 0; 0 |
| treecover2000 | C | Tree canopy cover in 2000 (%) | Hansen treecover2000; 0 where masked | – | Removed | – |
| treecover2000_250m | C | Tree cover, 250 m mean (%) | Focal mean (r = 250 m) of treecover2000 | – | Retained | 0; 0 |
| canopy_shade_proxy | C, L | Canopy shade index (0–1) | 0.5 × ndvi_mean + 0.5 × treecover2000 / 100 | – | Retained | 0; 0 |
| forest_loss_any | C | Any forest loss 2001–2024 (0/1) | Hansen lossyear > 0 | – | Retained | 0; 0 |
| years_since_loss | C | Years since forest loss | 2024 − lossyear (coding error, see Notes) | – | Removed | – |
| lc_type1 | C | Land-cover class (IGBP, 17 classes) | MCD12Q1 LC_Type1 | Categorical (dummy-coded) | Retained | 0 for all classes; 0 |

In the local GAM, canopy_shade_proxy had the largest effect (edf = 4.92, χ² = 161.8, p < 0.001).

**5. Auxiliary layers (exported in the stack, not used as predictors)**

| Code | Description | Derivation in GEE | Use |
| --- | --- | --- | --- |
| land | Land mask (1 = land) | MERIT Hydro upa not masked AND lc_type1 ≠ 17 (water) | Background sampling (10,000 random 30 m land cells) and masking of prediction maps |
| historical_template_index | Composite historical-template index | hydro_persistence_proxy + 0.05 × relief_1km + 0.2 × valley_depth_1km − 20 × tc_pr_ann_cv − 20 × tc_soil_ann_cv − 5 × tc_vpd_ann_sd (ad hoc weights) | Visualisation only |
| aspect_deg | Aspect (°) | ee.Terrain.products | Source of northness and eastness |
| slope_sd_300m | Focal SD of slope, r = 300 m (°) | As slope_sd_1km | Not used |
| merit_elv_m | MERIT Hydro elevation (m) | MERIT Hydro elv | Not used |
| water_change_abs | Absolute change in water occurrence (%) | JRC change_abs | Not used |
| lossyear | Year of forest loss (1–24 = 2001–2024) | Hansen lossyear | Source of forest_loss_any |
| ndvi_250m, ndvi_1km | NDVI focal means (r = 250 m, 1 km) | Focal mean of ndvi_mean | Not used |
| treecover2000_1km | Tree cover focal mean (r = 1 km) | Focal mean of treecover2000 | Not used |

**6. Notes**

**Common processing.**

1. Extent: bounding box 126.08–126.99° E, 33.06–33.66° N. All layers were exported from GEE at 30 m in EPSG:4326 (70 bands), so coarser datasets keep their native cell structure.
2. Focal statistics use circular kernels of the stated radius and skip masked pixels.
3. In R, upa_km2, hnd_m, dist2persistentwater_m and relief_1km were log1p-transformed. Within each training fold, missing values were replaced by training medians and predictors standardized to zero mean and unit variance; lc_type1 was dummy-coded.
4. Pre-screening was done separately within sets H and C on the full analytical dataset. For each pair with |r| ≥ 0.97, the variable with the higher mean |r| to the others was removed. Protected (never removed): elev_m and hydro_persistence_proxy (H); twi_proxy, cold_air_pooling, canopy_shade_proxy and solar_exposure_proxy (C). 24 of 40 H and 18 of 21 C variables were retained. Results with |r| ≥ 0.70 and VIF < 5 screening are shown in Fig. S1.
5. The local GAM used seven fixed smooth terms (L), regardless of pre-screening.

**Known limitations of the predictors.**

- Sets H and C differ mainly in spatial scale and assumed persistence. Neither is a direct palaeoenvironmental, demographic or genetic measure.
- valley_depth_300m equals −tpi_300m exactly, and each clim_stab_* variable is a sign-reversed copy of a variability metric. Pre-screening therefore removed them by construction.
- JRC Global Surface Water maps coastal sea as water, so dist2persistentwater_m measures distance to the coast or to inland persistent water, whichever is nearer.
- years_since_loss was computed as 2024 − lossyear, but lossyear is coded 1–24, so the variable is effectively binary. It was removed by pre-screening and did not enter any model.
- hydro_persistence_proxy, canopy_shade_proxy, cold_air_pooling, solar_exposure_proxy and historical_template_index use fixed weights chosen a priori, not estimated from data.
- cold_air_pooling is valley_depth_300m weighted by aspect. Its GAM smooth is therefore conditional on valley depth and should not be read alone.

**Data sources (GEE asset IDs).** NASA/NASADEM_HGT/001; MERIT/Hydro/v1_0_1; JRC/GSW1_4/GlobalSurfaceWater; IDAHO_EPSCOR/TERRACLIMATE; MODIS/061/MOD13Q1; MODIS/061/MCD12Q1; UMD/hansen/global_forest_change_2024_v1_12. Code: GEE extraction script and R analysis scripts in the Zenodo repository.
